# Mindfulness training for community-based psychotherapists: a feasibility study

**DOI:** 10.1186/s40814-022-01205-x

**Published:** 2022-12-09

**Authors:** James T. Sucich, Jeremy Lehrer, Vicki Breitbart, Kell N. Julliard

**Affiliations:** 1 Family Health Centers at NYU Langone, 514 49th Street, Brooklyn, NY 11220 USA; 2 Columbia University, New York City, USA; 3 Silver School of Social Work, New York University, New York City, USA

**Keywords:** Mindfulness training, Feasibility, Community mental health clinic, Psychotherapist

## Abstract

**Background:**

While mindfulness training’s feasibility has been assessed in many health care settings, the feasibility of teaching mindfulness to psychotherapists of various orientations for both self- and patient-care has not been explored. The objectives of this feasibility assessment were to determine the degree to which clinic psychotherapists were willing to complete a skills-based mindfulness training program; evaluate the acceptability of integrating mindfulness interventions into an urban community mental health clinic; examine the training’s influence on both personal mindfulness practice and integration into patient care; and explore the impact of a support group following the training.

**Methods:**

Data on six aspects of feasibility were gathered through quantitative surveys, semi-structured qualitative interviews, and group observation and feedback, analyzed using grounded theory.

**Results:**

Sixteen therapists and one administrator attended at least one session of this voluntary program and responded to the associated surveys. At 1-year post-training, 7 participants had attended one or more group support sessions, and 4 more than 50% of sessions. The following factors were identified as contributing to the training’s success: significant interest on the part of clinic staff to receive the training; diversity of the teaching staff, buy-in from clinic administration, provision of meditation scripts, role-play exercises, the variety of practices taught, and case presentations. Therapists indicated that the training helped them create a personal mindfulness practice, and several proceeded to integrate mindfulness into client sessions. A bi-weekly support group organized after the training encompassed group practice, discussion, case presentations, and information about trauma-sensitive mindfulness. Clinicians identified the following challenges to integrating mindfulness into sessions: lack of scripts in client languages other than English, the unacceptability of mindfulness to some clients’ religious beliefs, the lack of appropriateness for clients facing ongoing psychosocial crises, the lack of interest on the part of some clients, and the time constraints posed by brief therapy sessions.

**Conclusions:**

These findings indicate that such training may be feasible in community mental health settings given support from leadership and the presence of qualified facilitators within the organization. Adaptations to the training based on participant feedback can inform a larger scale trial that compares our protocol with another intervention in the treatment of a psychological disorder or condition identified by the participants as having responded favorably to the program.

## Key messages regarding feasibility

- Uncertainties regarding feasibility: questions prior to the study included (1) Would urban, clinic-based psychotherapists of various psychotherapeutic orientations be willing to complete a mindfulness training program during work hours, on their own time? (2) Could the skills-based mindfulness program be accepted and integrated into the clinic? (3) Would interested clinicians be willing to implement the skills with their patients? (4) Would a post-training support group encourage personal care and ongoing skill development, prevent skill decay and prepare interested participants to test the program against another psychological intervention in the treatment of a condition or disorder that they identified as having responded favorably to the program?
- Key findings: the findings support the feasibility of integrating a skills-based mindfulness program into a community mental health clinic. For a subset of clinicians, post-training support facilitated personal care and the willingness to implement the skills with patients.
- Implications of the findings: the findings of this feasibility study suggest that the program, with some modifications, can be compared to another psychological intervention in the treatment of a condition or disorder common to the clinic. Following another single-group study to solidify the format of the program and establish the best measures to assess achievement of its goals, an efficacy trial conducted at a single site would be modest but could inform the design of a larger randomized control trial (RTC) at multiple sites within the hospital system.

## Introduction

Mindfulness training has been shown to benefit the mental health and well-being of both behavioral health patients [1, 2, 3, 4, 5], and health professionals, including social workers [6, 7, 8, 9] and other psychotherapists [10, 11]. Therefore, training mental health professionals to practice mindfulness and bring these practices to their patients should result in benefit to both patient [12] and provider [10].

More specifically, the application of evidenced based practices in clinical settings has produced a burgeoning of support for mindfulness-based interventions (MBI) in the treatment of psychological, psychiatric, and physical conditions. Recent meta-analyses have confirmed the efficacy of MBIs for specific psychiatric disorders and related conditions including depression, chronic pain, smoking, and addiction [13, 14, 15] as well as preliminary evidence of efficacy for eating disorders, attention-deficit/hyperactivity disorder (ADHD), post-traumatic stress disorder (PTSD) and serious mental illness [16]. At our clinic, nearly 75% of active patients over a 9-month period had a primary diagnosis of depression and/or anxiety which indicates that MBIs would be applicable to a large number of our patients. MBIs also may have unique utility in addressing psychiatric comorbidity by targeting underlying core cognitive and affective processes across diagnoses [16].

Several training programs for psychotherapists exist using specific theoretical approaches, but these usually focus either on the provider as participant or on teaching the provider to use the approach in patient care. These approaches include Mindfulness-Based Stress Reduction (MBSR ) [16], Mindfulness-Based Cognitive Therapy (MBCT) [17], Acceptance and Commitment Therapy (ACT ) [18], and Dialectical Behavioral Therapy (DBT) [19]. Such training approaches are often costly, time consuming, and difficult to implement at community mental health clinics, which usually have restricted budgets and time for training [20]. In addition, these specific trainings may not appeal to therapists of different theoretical orientations and different stages of professional development [9, 10, 21].

In community mental health settings, with motivated and qualified staff from a variety of therapeutic backgrounds, a trans-theoretical or integrative approach to mindfulness training [22, 23, 24, 25] could be beneficial for psychotherapists and patients [26]. According to Dunn et al, [22] regardless of the theoretical orientation of the therapist, mindfulness integrated into client therapy can improve clients’ awareness during sessions, reduce their ruminative thinking, and increase their self-compassion, which in turn can foster resilience.

To our knowledge, the feasibility of teaching mindfulness to psychotherapists of various orientations for both self- and patient-care has not been assessed. Feasibility studies of mindfulness training were present in other disciplines [27, 28, 29], and the benefit of training to therapists’ personal practice is gaining increasing recognition [30].

### Aims

We conducted such a training of our staff at an urban federally qualified health center with a highly diverse patient population and concurrently gathered data to determine whether it met the criteria for feasibility for both the therapists’ benefit and the integration of mindfulness practices into patient care. Bowen et al. [31] have recommended testing the feasibility of an intervention when prior studies have successfully produced positive outcomes but in settings different than the one of interest. Following Bowen et al.’s [31] recommendations, this study examined a number of factors that can be considered foundational to more rigorous future research, including the interventions’ adaptability, practicality, integration, and implementation. An investigation of these dimensions is an initial step toward determining the effectiveness of mindfulness-based interventions in community centers.

The study’s first objective was to investigate the feasibility and acceptability of integrating mindfulness interventions into a community mental health clinic by training experienced psychotherapists (social workers and psychologists) in a skills-based program designed specifically for the study. The second objective was to examine the training program’s influence on the participants’ personal mindfulness practices. The third objective was to assess whether interested participants were willing integrate their skills into their clinical work. The fourth objective was to explore the impact of a post-training support group on the implementation process for both personal and patient care.

## Methods

### Setting

We conducted the mindfulness initiative at an urban, federally funded outpatient mental health clinic in an ethnically diverse, underserved community. The neighborhood was 40% Hispanic, 33% Asian, 23% white, 2% Black, and 2% multiracial (United States Census, 2017). Poverty in the clinic neighborhood was 1.5 times the rate in the state (14.1%) and more than 1.5 times the rate in the USA (13.4%) (US Census, 2017). At the time of the training, 32 social workers and psychologists provided therapy for patients. The average clinician’s caseload was 78 patients, and his or her average number of patient visits per month was 106 (Table 1).

**Table 1:** Demographics of therapists registering for the training (*n* = 16)

Characteristic	***n***	%
**Gender**		
Female	10	62%
Male	6	38%
**Ethnicity/race**		
Asian American	2	13%
Black	2	13%
Hispanic/Latinx	7	44%
White	5	31%
**Religious or spiritual orientation**		
Catholic	6	38%
Spiritual	3	19%
Secular humanist	2	13%
Other	1	6%
None	4	25%
**Highest degree**		
MSW	5	31%
Masters in psychology	3	19%
Doctorate or MD	8	50%
**Therapeutic orientation**^**a**^		
CBT	14	88%
Analytic/dynamic	7	44%
Interpersonal	7	44%
Humanistic/gestalt/existential	5	31%
Other	3	19%
**Previous mindfulness training**^**a**^		
Books/self-taught	11	68%
Yoga	8	50%
In context of psychotherapy training	4	25%
Meditation workshop/retreat taught by clinician	6	38%
Meditation workshop/retreat/taught by religious teacher	9	56%
Other meditation workshop/retreat	4	25%
Other	2	13%
None	2	13%

^a^Participants could respond to more than one

### Participants

Given the broad interest in mindfulness within the larger therapeutic community, we anticipated the program would appeal to a significant number of our clinicians. Because our aim was to recruit as many interested therapists as possible, we did not set specific goals for the number of participants recruited or retained. Similarly, although we encouraged participants to attend all four training sessions, we did not, for the study’s purposes, establish a minimum cut off for attendance.

### Program description and training

As an introduction to basic mindfulness practices (e.g., breath awareness, body scanning), participants were instructed to complete the 10 introductory sessions of Headspace, a highly rated smartphone-based mindfulness app shown to increase mindfulness [32]. A website was created specifically for the training as a forum for discussion, inquiry, and support and contained video recordings of the course instructors introducing themselves and explaining the training’s objectives.

The training was offered in four 90-min sessions held biweekly during April and May, 2018. Sessions were “off the clock” during lunch breaks. Drawing on the three facilitators’ training and experience, the program was designed to teach basic mindfulness practices that participants could do on their own and implement with their clients. Participants were asked to download a second mindfulness app, Insight Timer, on which two of the instructors posted guided meditations. Online modules introduced practices and concepts to accompany each training session.

One facilitator was a PhD psychologist and certified MBCT instructor who used mindfulness in her clinical work and collaborated on clinical trials involving mindfulness at NYU School of Medicine, where she developed mindfulness-based interventions for mental and physical health disorders. Another facilitator was head of clinical research at the health center and had taught the secular Joy of Living [3, 33] mindfulness training on this health care campus since 2011. The third facilitator was a PhD clinical psychologist who had completed the MBSR program and the three Joy of Living training levels and had certificate training in MBCT and ACT.

The training program incorporated elements of MBCT, MBSR, and the first two levels of the Joy of Living (awareness/mindfulness and loving kindness/compassion). Each session blended didactic instruction, experiential exercises, and group discussion. Written scripts of formal mindfulness practices were made available for in-session exercises and use between sessions (Table 2). Homework was assigned at the close of each session: participants were instructed to listen to guided meditations on Insight Timer and to post comments and questions on the course website. Following the third session, participants were asked to identify a client they thought could benefit from mindfulness and to outline the pros and cons of introducing them to mindfulness practice. They were also asked to use one of the scripts to practice guiding another course participant in a meditation.

**Table 2:** Mindfulness exercises taught during the 4-session training^a^

Mindfulness practice	Content	Session
Open presence	Let your mind rest without distraction, simply knowing that you are aware in this moment. Thoughts and sensations can come and go without the need to block or control them.	1
Mindfulness of sound	Bring awareness to the experience of listening to sound. Simply know that you are hearing sounds.	1
Mindfulness of body sensations	Bring awareness to the experience of any sensation of your choice present in your body. Simply know that you are feeling that sensation.	1
Body scan	Bring awareness to the sensations present in each part of your body. Start at the top of your head and move down sequentially to the bottom of your feet, or vice versa.	1
Mindful movement	In whatever position or activity you find yourself in, bring awareness to the experience of any movements that occur. Simply know that you are moving.	1
Mindfulness of walking	At whatever pace you are naturally walking, bring awareness to any aspect of the experience of walking. For beginners, pick something simple such as the feeling of each foot leaving the ground, moving through space, and touching the ground, or the swinging of your arms.	1
RAIN meditation	1. Recognize that an emotion or mood is present. 2. Accept that it is present, that you cannot magically make it disappear. 3. Investigate the feeling of the emotion in your body, the actual physical sensations associated with that emotion. 4. Note: describe the physical sensation with a brief phrase (such as “flushed face”) to sharpen your experience of the sensation. (Alternatively, this can stand for Non-identification, that you are not the emotion.)	2
Mindfulness of thoughts	Whether letting the mind rest or thinking intentionally, simply know that you are thinking while you are thinking.	2
Working with difficulty	Bring awareness to the body as a whole. Notice any challenging thoughts or emotions that arise. Allow them to remain in awareness. Notice any bodily sensations associated with the emotion. Notice where the sensations are strongest. Imagine your breath coming and going in this area to help you explore the feeling. Deepen your attitude of acceptance of the sensations. Soften and open to the sensations. Return to awareness of the breath if the emotion subsides.	2
Lead a partner in a focused-attention practice	This could be one of the practices related to sound, body sensations, body scan, movement, walking, or thoughts above.	2
Loving-kindness and compassion for oneself	Notice your wish to be happy and free from suffering (frame this wish in a way that feels right for you). Softly repeat the phrase, “May I have happiness and its causes.” Wish this sincerely and warmly to yourself. Feel free to create your own phrases such as, “May I be healthy/successful/have ease/be free from inner and outer harm” or imagine a warm light or loving hug enveloping yourself. After a while, repeat the phrase, “May I be free from suffering and its causes,” or create a similar phrase that is meaningful for you.	3, 4
Loving-kindness and compassion for others	Notice your wish to be happy and free from suffering (frame this wish in a way that feels right for you). Choose a loved one or neutral person or animal (whoever is easiest to love). Recognize that they too wish to be happy and free from suffering. Softly repeat the phrase, “May you have happiness and its causes.” Wish this sincerely and warmly to that being. Feel free to create your own phrases such as, “May you be healthy/successful/have ease/be free from inner and outer harm” or imagine a warm light or loving hug enveloping the person. After a while, repeat the phrase, “May you be free from suffering and its causes,” or create a phrase that is similarly meaningful.	3, 4
Lead a partner in the RAIN or “Working with difficulty” meditation		3, 4

^a^Participants were typically asked to practice the mindfulness techniques taught in a given session during the weeks following that session. The practices were adapted from the Joy of Living program [33] except for RAIN [15, 34] and Working with difficulty [35]

Sessions 1 and 2 focused on core practices, while sessions 3 and 4 focused on practices the facilitators believed were distinctly relevant to psychotherapists and their clients (Table 2). The practices Working with Difficult Emotions and RAIN focus on emotional experiences, while Loving Kindness and Compassion practice focuses on building empathy and compassion for self and others. The third session included a case study of a client who benefitted from mindfulness practices in her treatment for depression and chronic pain. Session 4 included the topic “Bringing Mindfulness Practices to Clients” with concrete examples. Video recordings of the meditation and instruction periods of each session were available for participants to review.

At the close of the final training session, participants were invited to attend a biweekly support group co-led by two of the facilitators. Conducted during the lunch hour, the group was a forum for questions about mindfulness principles and practices and was intended to foster continued practice both on one’s own and with clients. It offered guided meditations, case discussions, and instruction on mindfulness techniques. The group was conducted on site until March of 2020, when, due to the global pandemic, it was moved to a remote platform. At 1-year post-training, 7 participants had attended one or more group supervision sessions. Four participants had attended more than 50% of the sessions.

### Feasibility study design

This was a mixed methods feasibility study incorporating quantitative surveys, semi-structured qualitative interviews, and observation notes from the support group formed post-training. Because the study was conducted at a federally qualified health center the population of which is primarily poor and underserved, we used a feasibility study model designed for this purpose [31] which is oriented toward public health, disease prevention, and health promotion. The areas of focus within that model that were investigated included acceptability, demand, implementation, practicality, adaptation, and integration. The different sets of data mentioned above were used to corroborate each other and determine the feasibility of the program [36, 37].

### Quantitative data analysis

At the end of the fourth (final) training session, participants completed a written survey asking for general feedback about the entire course and whether they had practiced on their own and with clients. A 6-month post-training follow-up survey was administered online to all participants. The objectives of this survey were to determine whether participants were practicing mindfulness on their own and with clients and to identify the conditions that fostered or deterred ongoing practice. These surveys were created to satisfy face validity and were not previously validated. Percentages of responses were calculated for all variables to assess acceptability and integration and to enable adaptation per the feasibility model used [31].

### Qualitative data analysis

Post-training interviews were conducted several months after the training by the second author (JL), a research assistant not directly involved in the program’s implementation. These interviews were meant to capture an in-depth understanding of therapists’ experiences and challenges implementing mindfulness into their own practice and their therapy sessions. Utilizing grounded theory, the notes from the interviews were reviewed several times by one author (VB) using standard techniques as outlined in Miles and Huberman [38]. The material was annotated and coded by hand and analyzed for patterns, commonalities, and differences until themes emerged. The material with emerging themes was then presented to the other authors, discussed, questioned, and verified. The results were continuously compared, compiled, and synthesized with respect to the feasibility framework.

In addition to the interviews, one of the authors (JL) observed and took notes from the support group sessions for 3 months post-training. The notes were reviewed, annotated, and analyzed as above to assess themes related to the project’s feasibility.

This study was considered exempt from IRB review according to the IRB of record’s policies, and this was documented with the appropriate forms.

## Results

### Participants

Sixteen therapists responded to a clinic-wide invitation to receive training and attended at least one session of this voluntary program. Thirteen respondents were social workers and psychologists who, as a group, represented approximately 40% of the clinic’s direct service providers. Two participants were social work administrators and one was the medical director. Eight participants reported a doctorate or MD as their highest degree; five listed MSW, and three a Masters in Psychology. Of the 16 participants, 15 attended the first session, 12 the second and third sessions, and 10 the fourth session. Six therapists attended four sessions, six attended three sessions, two attended two sessions, and two attended one session. The majority of the participants were people of color with different religious orientations and therapeutic training. Registered participants completed an online-questionnaire prior to the initiation of the training. Only two had no previous meditation training; others were either self-taught or had different previous training in meditation (Table 1).

Data reported below are from qualitative interviews and support group session notes unless otherwise noted. The diversity of the trainers, the support of clinic administration, the experiential aspects of the training along with its clinical relevance and the importance of therapist personal practice were all regarded positively. Therapists identified challenges such as time constraints and client receptivity, and integration of mindfulness into client sessions was highly variable.

### Elements of the skills-based training

Therapists identified several factors that increased their receptivity to the training. For some, the diversity of the teaching staff had a positive impact. “As a woman of color,” one clinician said, “I was glad that there was a person of color presenting, that it wasn’t just two white men”.

Affirmation from the clinic’s administrative staff also fostered receptivity. The medical director supported the program’s development and participated in the training. His presence was noted by some participants as supporting their desire to further develop their clinical skills. “It is important that leadership was involved to sponsor and validate [the training]… and he wants the center to value mindfulness.”

Experiential aspects of the training, including the meditation scripts and the dyad and triad role-play exercises, fostered skill development. Participants responded favorably to the blending of instruction, practice, discussion, and exercises and to the interactive nature of the training. One therapist stated, “I definitely thought the experiential pieces [that] we were getting to do were really helpful. When I was on the receiving end of getting a training – that helps solidify and give the recipient a hands-on experience.”

Participants described the program scripts and recorded meditations as beneficial, tangible, and relevant to their clinical work. “I liked the resources; how accessible they were ….so we could look it over, and practice leading a partner into a meditation.”

A number of participants noted that their learning was enhanced by the case presentation of a woman who benefitted over several sessions from mindfulness practices adapted to her treatment for depression and pain. A number of participants commented on the breadth of practices and expressed an appreciation for the instructors’ willingness to provide therapeutic and theoretical context for the curriculum. They felt the curriculum explained the scope and application of mindfulness practices as well as the integration of mindfulness with other therapeutic modalities, such as DBT and trauma-focused CBT.

In the evaluation form administered at the end of the last training session, participants commented favorably on the clarity of the instruction, the accepting and encouraging atmosphere, and the use of guided meditations. Several wanted further sessions and ongoing reinforcement of the material as well as continued access to the meditations and resources on the course webpage.

### Building therapists’ personal practice

Therapists spontaneously commented on their personal benefit from learning about and practicing mindfulness. One therapist said, “I do [mindfulness] sporadically when my stress levels become really high...when I need to ground.” Some perceived how the benefits of mindfulness spilled over from therapist to patient; for instance, one therapist commented, “I think mindfulness is an incredible tool to be able to model for somebody else how to stay present and navigate difficult moments.” One of the perceived personal benefits was to enhance their skill as a clinician: “I think mindfulness makes a person a better clinician in that you’re more sensitive, more compassionate, with yourself and with another person, more aware of what is happening in the moment. Being a clinician is really hard, we all struggle in the moment with all kinds of difficulties, our own challenges when we’re engaging with a patient.”

Clinicians who reported having a personal mindfulness practice said their experience prepared them to introduce mindfulness to their clients. They felt that on-going practice would make them more effective in guiding clients during sessions. One clinician commented, “There are a couple of reasons why personal practice is important…if you don’t practice yourself, it’s hard to tell other people to practice and how to problem-solve.” A second clinician emphasized how ongoing practice boosts confidence. She said, “Individually, it gives me more confidence if I’m practicing regularly the mindfulness strategies that we learned.”

In the 6-month follow-up survey that was sent to therapists who completed the training, 11 responded, and 9 stated that the training workshops had influenced their practice. Compared to the pre-training survey, the 6-month post-training survey revealed a moderate increase in the number of therapists who practiced meditation on their own and in their practice with clients. (Table 3).

**Table 3:** Comparison of pre-course and 6-month follow-up surveys (*n* = 11)

Practice question	Never		Rarely		Occasionally		Frequently		Very frequently	
Therapists personal practice	*N*	%	*N*	%	*N*	%	*N*	%	*N*	%
Practiced meditation **before** training^a^	3	18%	4	24%	4	24%	4	24%	2	12%
Practiced meditation **after** training^b^	0	0%	3	30%	3	30%	3	30%	1	10%
Used meditation with clients										
**Before** training^a^	4	24%	8	47%	4	24%	1	6%	0	0%
**After** training	2	18%	2	18%	5	45%	2	18%	0	0%

^a^17 responses to the pre-course survey (one respondent did not attend the training)

^b^10 respondents to this follow-up survey question

### Integrating mindfulness into clinical practice

In the post-training interviews, clinicians remarked on several challenges they faced integrating mindfulness practices into their clinical work. Some bilingual participants commented on the lack of scripts in languages other than English. They felt the availability of scripts in Spanish and Chinese, the predominant languages of their clients, would facilitate implementation. A number of therapists felt client receptivity was possible if the introduction to mindfulness was culturally sensitive. One therapist’s strategy was to avoid the word “mindfulness” and refer to the practices as “tools” that could be used to address a specific issue, for instance, improving one’s relationship with a spouse. One therapist who worked with adolescents stated, “Helping them understand what it is, and doing it again and again, it’s a slow process. I have never had a kid that didn’t benefit from it.”

Clinicians questioned the acceptability and practicality of integrating mindfulness into the clinic setting. Some said mindfulness for clients facing ongoing psychosocial crises, trauma, and severe mental illnesses seemed inappropriate. In the follow-up survey, 6 therapists of 11 identified that some clients were not interested in mindfulness, and two that mindfulness practice may not be appropriate for some clients. “My clients don’t really want to focus on mindfulness if they have so many things they want to tell me… With our particular clients, sometimes you’re able to use mindfulness, but sometimes you have to address crises…. If they’re in crisis, you have to focus on problem-solving.… With mindfulness and meditation,... we’re asking them to practice awareness and attention regulation skills that are somewhat challenging to do.”

In both the interviews and the 6-month follow-up survey, time constraints were mentioned as a major impediment for integrating mindfulness into practice. Clinicians commented that that their large caseloads and the abbreviated length of the typical psychotherapy session (30 min) did not allow sufficient time with each client to teach mindfulness effectively. In the follow-up survey, 9 of 11 (82%) participants stated that insufficient time was the biggest obstacle to incorporating mindfulness into their practice.

Therapists’ lack of confidence in their own practice was a barrier to implementation with clients. In the follow-up survey, 5 of 11 believed they lacked confidence to do the practice on their own or with clients, three therapists identified their lack of a personal practice as a challenge to bringing mindfulness into sessions, and three did not feel comfortable leading mindfulness practices with clients. Therapists who practiced on their own and introduced mindfulness into clinical sessions, however, reported benefits.

In the 6-month follow-up survey, 9 of 11 respondents indicated that they were using mindfulness in sessions. Before the training, 29% reported that had occasionally or frequently used mindfulness with clients; after the training 64% of the respondents said they did (Table 3).

Therapists were selective about which patients they chose for mindfulness practice. “I’ve been incorporating a lot of the scripted meditations. I’m using it with a handful of patients. Meditating with the breath, body scan, body sensations...I’ve been working with thoughts, with patients who are anxious about pain.”

**Fig. 1:**
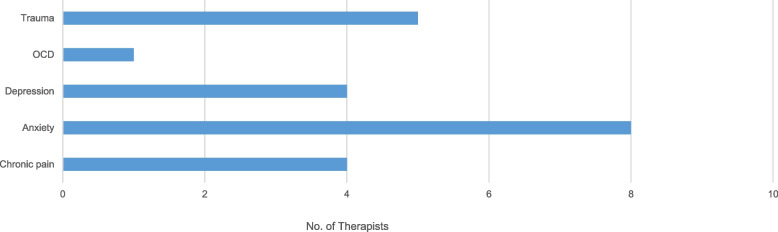
Therapist perceptions of client issues^a^ for which mindfulness was most helpful in session (*n* = 11). ^a^OCD = obsessive-compulsive disorder. Two therapist respondents were not using mindfulness in session. 1 Miles, MB Huberman, AM

In the 6-month follow-up survey, therapists reported using mindfulness most often with anxious clients or those with a history of trauma (Fig. 1: Client issues for which mindfulness was perceived by therapists to be most helpful).

In the interviews, therapists stated that the busyness of the clinic and the diversity of the clients were obstacles but that the support group assisted integration. One therapist stated, “There are so many nuances to teaching mindfulness and bringing it into a clinical setting that it would take more…. It’s a life approach really.”

Integration and acceptability of meditation with clients were also explored in the support group. Seven therapists attended the support group regularly. Some reported obstacles when working with Hispanic clients who viewed meditation and mindfulness as counter to the Christian faith. Therapists working with people of color, both adults and teens, noted that mindfulness and meditation were sometimes seen as practices of privileged white people that had no relevance to them. Clinicians noted that support group sessions—which encompassed group practice, discussion, case presentations, and information about trauma-sensitive mindfulness [39]—helped them to continue practicing and learn more about the applications of mindfulness. All seven respondents who attended the support group found the sessions provided opportunities to share experiences and supported their own practices. Of those who had not attended these sessions, most stated in the 6-month online survey that they did not have time in their busy schedules; one stated that the meeting time was not convenient, and one was not implementing mindfulness interventions.

## Discussion

Our findings support the feasibility of integrating mindfulness into a community mental health clinic by developing a skills-based training with examples and scripts for therapists to use with clients. The training did increase the personal mindfulness practice of interested therapists and provided support for them to implement mindfulness practice into therapeutic sessions with clients. According to the aspects of feasibility identified by Bowen et al. [31], our findings indicated that the program was acceptable to a subset of therapists and to clinic leaders; practical and integrated in that it did become part of the practice of the organization; and efficacious in that it was perceived as being beneficial to both the quality of life and clinical practice of the therapists who adopted it (Table 4). Following Bowen et al.’s [31] recommendations, this study’s findings, including the intervention’s adaptability, practicality, integration, and implementation, can be considered foundational to a more rigorous future research project that compares the program’s efficacy to that of another similar intervention.

**Table 4:** Findings related to feasibility as categorized by Bowen et al.^a^ (Bowen et al. 2009)

Dimension of feasibility	Findings from present study
Acceptability to individual recipients	● Post-training interviews indicated that training was acceptable to all who participated
Demand for training	● Almost 40% of clinic therapists attended training voluntarily ● Attendance was consistent at both training and support group
Implementation as planned	● Training was carried out in time frame as planned ● Ongoing support group was added and was continuing at 1 year post-training
Practicality of training delivery	● Training was given support from leadership and space ● Content was valuable enough to therapists that attendance was regular ● Presence of trainers within the organization was essential
Adaptation of training contents to new situation	● During training, feedback loops were established so that it could be modified to meet participant needs and perspectives ● Support group was created to provide ongoing guidance and encouragement
Integration into existing infrastructure	● Post-training survey indicated an increase in integrating meditation into therapists’ personal practice and sessions with clients ● Biweekly post-training support group provided regular and visible evidence of integration of mindfulness practice

^a^Expansion and limited-efficacy testing were not assessed

### Comparison with findings in the literature

Published feasibility studies related to the mindfulness training of health professionals often focused on the benefits to the professionals themselves. Luberto et al. [40] reported the feasibility, acceptability, and effectiveness of MBCT for hospital employees, who generally found the program valuable. These authors identified significant decreases in stress and burnout pre- to post-intervention. Their session attendance declined to 54% by session 4, however. In our initiative, attendance was relatively constant, although no session was attended by all who registered.

Amutio et al. [27] investigated the acceptability and effectiveness of MBSR plus a 10-month maintenance phase consisting of a monthly meeting. As with our study, these authors, who offered their program to physicians, found that the maintenance phase enhanced the benefits provided by mindfulness as a result of the initial training.

In a study of the feasibility of modified mindfulness-based cognitive therapy training to reduce therapist stress, Marx et al. [41] found increases in mindfulness, improvements in wellbeing, and positive changes to work life. Their retention during the formal training period was similar to ours—42 of 47 therapists attended at least 4 of the 8 sessions. Compared to our study, they did not reinforce integration with a follow-up support group. Trowbridge et al. [42] implemented a two-day MBSR training for pediatric medical social workers. While the ProQOL subscale of Burnout and the Perceived Stress Scale-10 did not decrease significantly (a trend toward decrease with a medium effect size was present), participants did demonstrate a significant decrease on the ProQOL Secondary Traumatic Stress Subscale, a measurement of therapist stress related to working with clients who have traumatic stress. MAAS scores (measure of participant mindfulness) also significantly increased. This accords with the qualitative reports of our participants, such as being more grounded, more easily navigating difficult moments, and becoming a better clinician.

In a study of general practitioners in the UK National Health Service, [28] Hamilton-West reported that physicians found many benefits to a mindfulness CBT course, including gaining new understanding and learning practical techniques to manage stress. They identified challenges in developing a mindfulness practice, such as finding time, making a new habit, and overcoming negative preconceptions. Many reported feeling more relaxed and experiencing increased compassion for self and others. All of these studies, as does this one, support the value of mindfulness training for therapists’ self-care.

Various studies support the feasibility of mindfulness training that extends beyond basic introductory courses. Ruijgrok-Lupton et al. [43] reported that the participants in courses of MBSR teachers who had an additional year of training recorded greater well-being and lower perceived stress. This reinforces the value of the ongoing support group we offered therapists after the formal four-session course. The findings of Ruijgrok-Lupton et al. [43] suggest that ongoing therapist training and support in clinical settings are likely to translate into gains in patient care. Waelde et al. [21] reported that fewer than half of their 116 trauma therapist respondents maintained a personal meditation practice, and 18% had received no training at all. This suggests a need for trainings and peer support groups similar to ours.

In addition to reinforcing therapist and patient care, training following implementation is important if the efficacy of our program (as applied to patient care) is to be adequately evaluated in a future study. The discontinuation of practice is the most frequently observed obstacle to mindfulness in studies of healthcare workers [44]. For instance, a recent feasibility study of a mindfulness program for U.K. physicians found that despite significant interest in the program, completion rates were low [45]. Similarly, a recent randomized control trial of a brief mindfulness training during work hours was found to be feasible but post-intervention improvements in self-care were not maintained at a 13-week follow-up [46]. The authors noted that work obligations and shift changes were two reasons for non-attendance, and while a reduction in stress levels was achieved, it is impossible to know whether these findings and the skills taught were maintained beyond the follow-up period.

Our extended support group therefore served the additional functions of bolstering participant retention and offsetting skill decay. A long-term approach to skill acquisition is supported by studies that demonstrate the influence of temporal sequencing and continuous practice on change [46, 47]. By offering recurring opportunities to practice, the support group reinforces skill development, fosters confidence and initiates discussions about the most effective ways to bring mindfulness to patients. The group will also allow the trainers to assess the readiness of interested participants to take part in a next phase assessment of the program’s efficacy in the treatment of patients.

### Findings relevant to other community behavioral health clinics

Our experience assessing the feasibility of this program generated insights that might be useful for other community mental health centers on limited budgets that wish to bring mindfulness to their patients and staff. First, support by organizational leadership was essential in allocating time for therapists to receive the initial training. The clinic director attended the training, which gave it credibility and importance that it might not otherwise have had. This leadership buy-in and the hierarchical collaboration it fostered are consistent with studies that show such organizational qualities are instrumental to successful program implementation [48, 49].

Even so, time constraints posed significant challenges to therapists being able to attend the training and to incorporate mindfulness appropriately into brief therapy sessions and with patients in crisis. Addressing these challenges and others, such as the contraindications of mindfulness, is a continuous aim of the ongoing support group.

The presence of qualified mindfulness trainers within the organization enabled the training itself and the post-training support group to take place without the need for extra funds. We were fortunate to be able to bring in outside expertise that enhanced diversity, which was appreciated by participants. Providing meditation scripts and key concepts in the languages of the patients served by the clinic would have been helpful for both the training and the support group.

We found it advisable to organize the ongoing support group to encourage the personal and professional application of the practices and principles taught during the formal training. The discussions and feedback in group sessions suggest that motivated therapists, when afforded consistent opportunities, will repeat practices, ask questions, dig deeper into the conceptual foundations of mindfulness, and explore case material in order to integrate mindfulness into patient care and their own lives. It must be emphasized that the support group is voluntary; participants attend on their own time. It is possible that attendance would be greater were the group part of an approved clinic in-service program, for which therapists receive release time. Indeed, institutional support through release time and funding is likely to promote the feasibility of mindfulness programs [45]

As indicated above, integrating mindfulness-based strategies into routine mental health care could improve the mental health of both provider and client. This would be best implemented in a way that can engage marginalized communities in mindfulness-based practices and strategies that are both culturally sensitive and responsive.

Our overall findings suggest a larger efficacy trial once the final format of the program is solidified and measures of the achievement of its goals are selected is a reasonable undertaking, one in which the mindfulness practices taught can be further refined and then formally compared to another psychological intervention for a disorder or condition common to the clinic and reported by the participants to have responded favorably to the program.

### Limitations

The limitations of this study are those typical to most feasibility studies. First, because the program was a collaborative effort by teachers with diverse training backgrounds, the program went through a number of changes, both before and during its implementation. The same was true of the concepts guiding post-training follow-up sessions. A lack of generalizability of the findings to other settings was therefore anticipated. Changes in program structure and content were made depending on participant responses, which may have been unique to our particular clinic. As with any mindfulness training program, the actual program content depended on the particular background and expertise of the trainers, which in this case was intended to be eclectic. Thus, the training content, as reported here (Table 2) would be difficult to duplicate. However, aspects of the protocol (e.g., meditation scripts, recordings of guided meditations) can be easily replicated or refined for a second phase study. A further limitation of the study was the small number of participants.

This research initiative lacked standardized measures of changes related to mindfulness practice in both therapists and patients. We relied on simple surveys and semi-structured interviews as a way of assessing feasibility and capturing the perspectives of participants. The reported findings can inform a pre-post efficacy trial that employs validated scales. Valid and reliable measurement of mindfulness can serve as one component of a comprehensive assessment of a participant’s readiness to implement the program with patients. Depending on the psychological disorder or condition targeted, standardized instruments of symptomatology can be employed to assess patients.

In light of the above discussion, we cannot quantify the cost versus benefit of this program. However, the cost was small, and participants indicated through surveys, interviews, and ongoing participation that the program was valuable for both themselves and their patients.

Based on the above findings, which suggest that this approach is feasible, the next step in researching this issue could consist of a more rigorous single group design to determine the best ways of quantifying outcomes. Diaries could be used to record therapists’ and clients’ perceptions of the benefits and challenges of mindfulness both in and out of session as well as their consistency of practice, with both quantitative and qualitative analyses of these data. Given the research into the benefits of mindfulness, such a study would not seek to replicate these findings but would focus more on measuring the extent to which culturally sensitive and linguistically appropriate mindfulness practices can be integrated into patient care.

## Conclusion

According to the criteria of Bowen et al. [31] (Table 4), given motivated staff, supportive leadership, and qualified internal trainers, basic cross-theory mindfulness training was feasible in this community mental health setting, improved therapists’ skills, and increased the implementation of mindfulness into patient care. The findings support a next phase efficacy trial following another single-group study to solidify the format of the program and establish the best measures to assess achievement of its goals.

## Data Availability

All data used and analyzed for this study are available from the corresponding author upon reasonable request.

## Abbreviations

ACT: Acceptance and commitment therapy
DBT: Dialectical behavior therapy
MBCT: Mindfulness-based cognitive therapy
MBI: Mindfulness-based intervention
MBSR: Mindfulness-based stress reduction
RCT: Randomized control trial
